# *Lactobacillus rhamnosus GG* and *Bifidobacterium longum* Attenuate Lung Injury and Inflammatory Response in Experimental Sepsis

**DOI:** 10.1371/journal.pone.0097861

**Published:** 2014-05-15

**Authors:** Ludmila Khailova, Benjamin Petrie, Christine H. Baird, Jessica A. Dominguez Rieg, Paul E. Wischmeyer

**Affiliations:** Department of Anesthesiology, University of Colorado School of Medicine, Aurora, Colorado, United States of America; Oklahoma State University, United States of America

## Abstract

**Introduction:**

Probiotic use to prevent nosocomial gastrointestinal and potentially respiratory tract infections in critical care has shown great promise in recent clinical trials of adult and pediatric patients. Despite well-documented benefits of probiotic use in intestinal disorders, the potential for probiotic treatment to reduce lung injury following infection and shock has not been well explored.

**Objective:**

Evaluate if *Lactobacillus rhamnosus* GG (LGG) or *Bifidobacterium longum* (BL) treatment in a weanling mouse model of cecal ligation and puncture (CLP) peritonitis will protect against lung injury.

**Methods:**

3 week-old FVB/N mice were orally gavaged with 200 µl of either LGG, BL or sterile water (vehicle) immediately prior to CLP. Mice were euthanized at 24 h. Lung injury was evaluated via histology and lung neutrophil infiltration was evaluated by myeloperoxidase (MPO) staining. mRNA levels of IL-6, TNF-α, MyD88, TLR-4, TLR-2, NFΚB (p50/p105) and Cox-2 in the lung analyzed via real-time PCR. TNF-α and IL-6 in lung was analyzed via ELISA.

**Results:**

LGG and BL treatment significantly improved lung injury following experimental infection and sepsis and lung neutrophil infiltration was significantly lower than in untreated septic mice. Lung mRNA and protein levels of IL-6 and TNF-α and gene expression of Cox-2 were also significantly reduced in mice receiving LGG or BL treatment. Gene expression of TLR-2, MyD88 and NFΚB (p50/p105) was significantly increased in septic mice compared to shams and decreased in the lung of mice receiving LGG or BL while TLR-4 levels remained unchanged.

**Conclusions:**

Treatment with LGG and BL can reduce lung injury following experimental infection and sepsis and is associated with reduced lung inflammatory cell infiltrate and decreased markers of lung inflammatory response. Probiotic therapy may be a promising intervention to improve clinical lung injury following systemic infection and sepsis.

## Introduction

Sepsis is a leading cause of death in infants and children despite the advances in medical and ICU care. Over 42,000 cases of severe sepsis are reported each year in the United States alone and millions are thought to occur worldwide [1]. Over 1 million deaths worldwide are associated with sepsis within the neonatal population [2], [3]. Low birth weight infants are particularly at risk, where the mortality is reported to be ∼50% [4]. Further the neonates who survive sepsis and septic shock continue to face substantial long term adverse effects [5]. Pediatric patients diagnosed with pneumonia or sepsis are also susceptible to acute lung injury or acute respiratory distress syndrome leading to a mortality rate of ∼25% [6], [7].

Critical illness and ICU care (broad spectrum antibiotics, poor nutrition deliver etc) creates a hostile environment in the gut and alter the microflora tilting the balance to favor pathogens [8]. Probiotics are living nonpathogenic bacteria colonizing intestine and providing benefit to the host with the potential to normalize the altered intestinal flora [9]. The use of probiotics in prevention of nosocomial gastrointestinal and respiratory tract infections in critical care has increased over the last few years and results from a growing number of randomized controlled trials within the adult and pediatric populations suggest their use as a promising treatment [10], [11], [12], [13]. The need for alternative, non-antimicrobial interventions for prevention of infection in an age of increasing antimicrobial resistance also make probiotics a promising strategy. Specifically, lactobacilli and bifidobacteria alone or in combination are the most frequently used strains in the treatment of various gastrointestinal disorders [14], [15], [16] or as therapy for different clinical conditions including antibiotic associated diarrhea [17], acute pancreatitis [18], ventilator associated pneumonia [11], [12], [19], sepsis and postoperative infections [20], [21].

Although probiotics are showing promise as an effective therapy in a growing number of illnesses, the mechanisms of their action are complex and still elusive [22]. Based on the results from several *in vivo* and *in vitro* studies, probiotics are able to decrease apoptosis in intestinal epithelial cells [23], [24], [25], [26], improve intestinal integrity [27], [28], [29], [30], prevent bacterial translocation [30], [31], reduce the overgrowth of pathogenic bacteria and suppress cytokine production [32], [33], [34], [35].

Despite the benefits of probiotic use in intestinal disorders, the effects of probiotic treatment to protect against lung injury following infection and sepsis are not well understood. We have recently shown the benefits of *Lactobacillus rhamnosus* GG (LGG) and *Bifidobacterium longum* (BL) on improved survival and intestinal homeostasis in weanling mouse model of cecal ligation and puncture (CLP) [36]. CLP is an experimental model of shock that mimics the pathology of sepsis occurring in the ICU patients [37]. Toll like receptors (TLRs) are pattern recognition receptors involved in the initial steps of signaling pathway leading to multiple organ failure in sepsis. TLRs bind to cell-wall components which activates nuclear factor (NF)-ΚB/IΚB system resulting in release of pro-inflammatory cytokines [38]. In addition to cytokines, pathogens activating TLRs were also reported to induce Cox-2 expression [39], [40].

In this study we hypothesized that LGG and BL will also have a protective effect against lung injury and will decrease the inflammatory response in the lungs potentially via the TLR/Myd88 pathway.

## Methods

### Probiotic treatment and septic peritonitis model

The animal protocol used in these studies was approved by the Institutional Animal Care and Use Committee of the University of Colorado Anschutz Medical Campus. Briefly, 3 weeks old FVB/N mice were orally gavaged with 200 µl of either LGG (1×10^9^ CFU/ml), BL (1×10^7^ CFU/ml), or sterile water (vehicle) immediately prior to initiation of the cecal ligation and puncture (CLP) procedure [41]. Briefly, a small midline abdominal incision was made, the cecum was ligated just distal to the ileocecal valve, and was then punctured twice with a 23-gauge needle. The cecum was squeezed to extrude a small amount of stool, replaced in the abdomen, and the peritoneum and skin were closed in layers. Sham mice were treated identically except the cecum was neither ligated nor punctured. All mice received 1.0 ml normal saline subcutaneously after the surgery to compensate for fluid loss. Mice received a single dose of probiotics prior to tissue collection. Animals were euthanized at 24 h.

### *Lactobacillus rhamnosus GG* and *Bifidobacterium longum* culture

LGG (ATCC, Manassas, VA) was incubated in MRS broth (BD, Sparks, MD) for 24 hours at 37°C and 5% CO_2_. BL (ATCC, Manassas, VA) was cultured in Trypticase soy broth (BD, Sparks, MD) for 72 hours in an anaerobic chamber at 37°C. A_600_ was measured to determine the number of colony forming units (CFU) per 1 ml. BL and LGG were pelleted from the broth (10,000 rpm; 10 min) and resuspended in distilled water.

### Immunohistology

Lung tissue was collected from each animal at 24 h and fixed overnight in 10% formalin, paraffin-embedded, and sectioned at 4–6 µm. Serial sections were stained with hematoxylin-eosin (H&E) and evaluated for severity of lung injury by blinded evaluator using a grading scale from 0 (no abnormality) to 4 (severe lung injury) as described previously [42].

Neutrophil infiltration into the lungs was evaluated by staining for myeloperoxidase (MPO). After deparaffinization and rehydration, sections were blocked with 1.5% rabbit serum (Vector Laboratories, Burlingame, CA) in phosphate-buffered saline for 30 min, then incubated with goat polyclonal MPO (1∶50; R&D Systems, Minneapolis, MN) antibody for 1 hour, washed with phosphate-buffered saline, and incubated with rabbit anti-goat biotinylated secondary antibody (Vector Laboratories) for 30 min. Vectastain Elite ABC reagent (Vector Laboratories) was then applied, followed by diaminobenzidine as substrate. Sections were counterstained with hematoxylin, dehydrated and cover-slipped. MPO positive cells were quantified in 10 random high-power fields per section. All counting was performed by a blinded evaluator.

### RNA Preparation, RT, and Real-Time PCR

Total RNA was isolated from lung tissue (snap frozen in liquid N_2_, collected at 24 h) using the RNeasy Plus Mini Kit (Qiagen, Santa Clarita, CA) as described in the manufacturer's protocol. RNA concentrations were quantified at 260 nm, and the purity and integrity were determined using a NanoDrop. RT and real-time PCR assays were performed to quantify steady-state mRNA levels of IL-6, TNF-α, MyD88, TLR-4, TLR-2, NFΚB (p50/p105) and Cox-2. cDNA was synthesized from 0.2 µg of total RNA. Predeveloped TaqMan primers and probes (Applied Biosystems) were used for detection. Reporter dye emission was detected by an automated sequence detector combined with ABI Prism 7300 Real Time PCR System (Applied Biosystems). Real-time PCR quantification was performed with TaqMan GAPDH controls and relative mRNA expression calculated using the 2^−ΔΔCT^ method [43].

### IL-6 and TNF-α protein analysis in the lung tissue

Lung tissue was harvested and frozen immediately in liquid nitrogen. Samples were homogenized with a hand-held homogenizer in a 5× volume of ice-cold homogenization buffer (Tris HCl, 50 mm; pH, 7.4; NaCl, 100 mm; EDTA, 10 mm; Triton X-100, 0.5%) with added protease inhibitors (Roche Diagnostics, Mannheim, Germany). The homogenates were centrifuged at 10,000 rpm for 5 min at 4°C and the supernatant was collected. Total protein concentration was quantified using the Bradford protein assay. Enzyme-linked immunosorbent assay (ELISA) (R&D Systems, Mineapolis, MN) was used to determine the concentrations of TNF-α and IL-6 in lung tissue homogenates according to the manufacturer's instructions.

### Statistics

Comparisons were performed with t test analysis (unpaired, two-tailed). To analyze the bacterial culture results, 2-tailed NPar, Mann-Whitney Test was used. No measurements or animals were lost to observation or missing in the analysis. Data were analyzed using Prism 4.0 (GraphPad Software, San Diego, CA) and reported as means ± SE. A p value≤0.05 was considered to be statistically significant.

## Results

### Probiotics improve lung injury and decrease the neutrophil infiltration during sepsis

**Figure 1:**
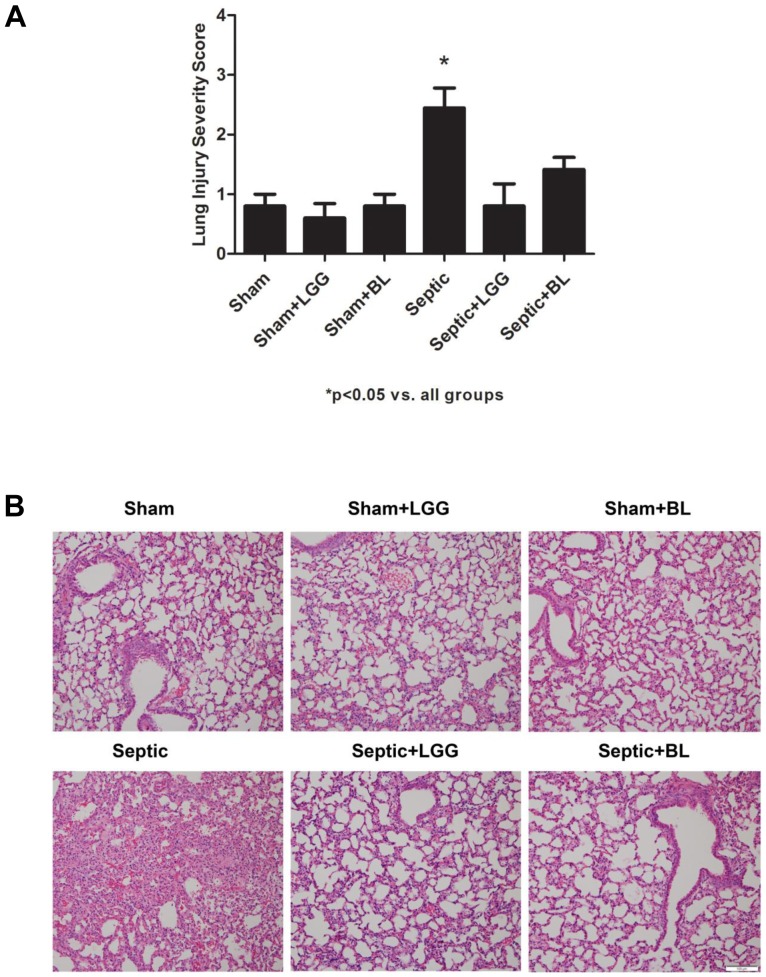
Probiotics improve lung pathology 24 hours post CLP. (A) The severity of pneumonia (from 0: no abnormality to 4: severe lung injury) was significantly reduced in the lungs of mice treated with LGG and BL (P<0.05). (B) Representative H&E stained sections of lung are shown. Original magnification ×100. Shams n = 4 per group, Septic, Septic+LGG, Septic+BL n = 5–8 per group.

**Figure 2:**
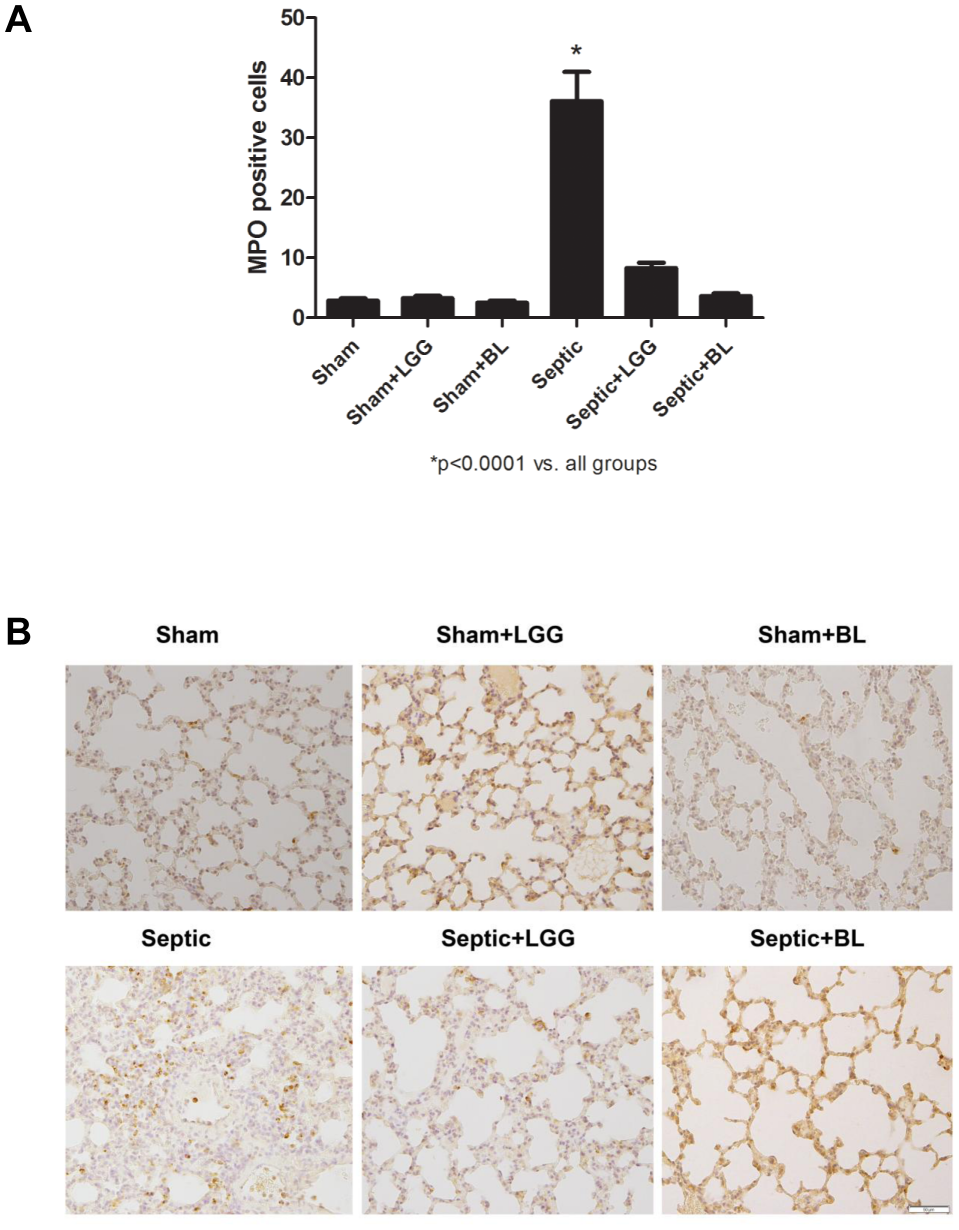
Probiotics decrease neutrophil infiltration in the lung 24 hours post CLP. (A) Number of MPO positive cells in the lungs of septic mice were significantly increased compared to shams. LGG or BL treatment normalized these levels (P<0.0001). (B) Representative MPO stained sections of lung are shown. Original magnification ×200. Shams n = 4 per group, Septic, Septic+LGG, Septic+BL n = 6 per group.

We have previously shown in this model that probiotic treatment with LGG or BL can improve survival following CLP [36]. In this study we hypothesized this may be associated with or be related to reduction in lung injury. Thus, the effect of probiotic treatment on lung pathology was assessed. Sepsis led to marked histological injury 24 hours after CLP surgery in septic animals. This injury was significantly improved in septic animals treated with LGG or BL (Figure 1A, B). Sepsis-mediated lung injury was associated with a significantly higher number of infiltrating neutrophils, represented by the number of MPO positive cells in the septic animals when compared to shams (P<0.0001). Treatment with LGG and BL normalized (P<0.0001) the number of MPO positive cells in the lungs to that observed in sham mice (Figure 2A, B).

### Probiotics attenuate proinflammatory cytokine release in the lung after sepsis

**Figure 3:**
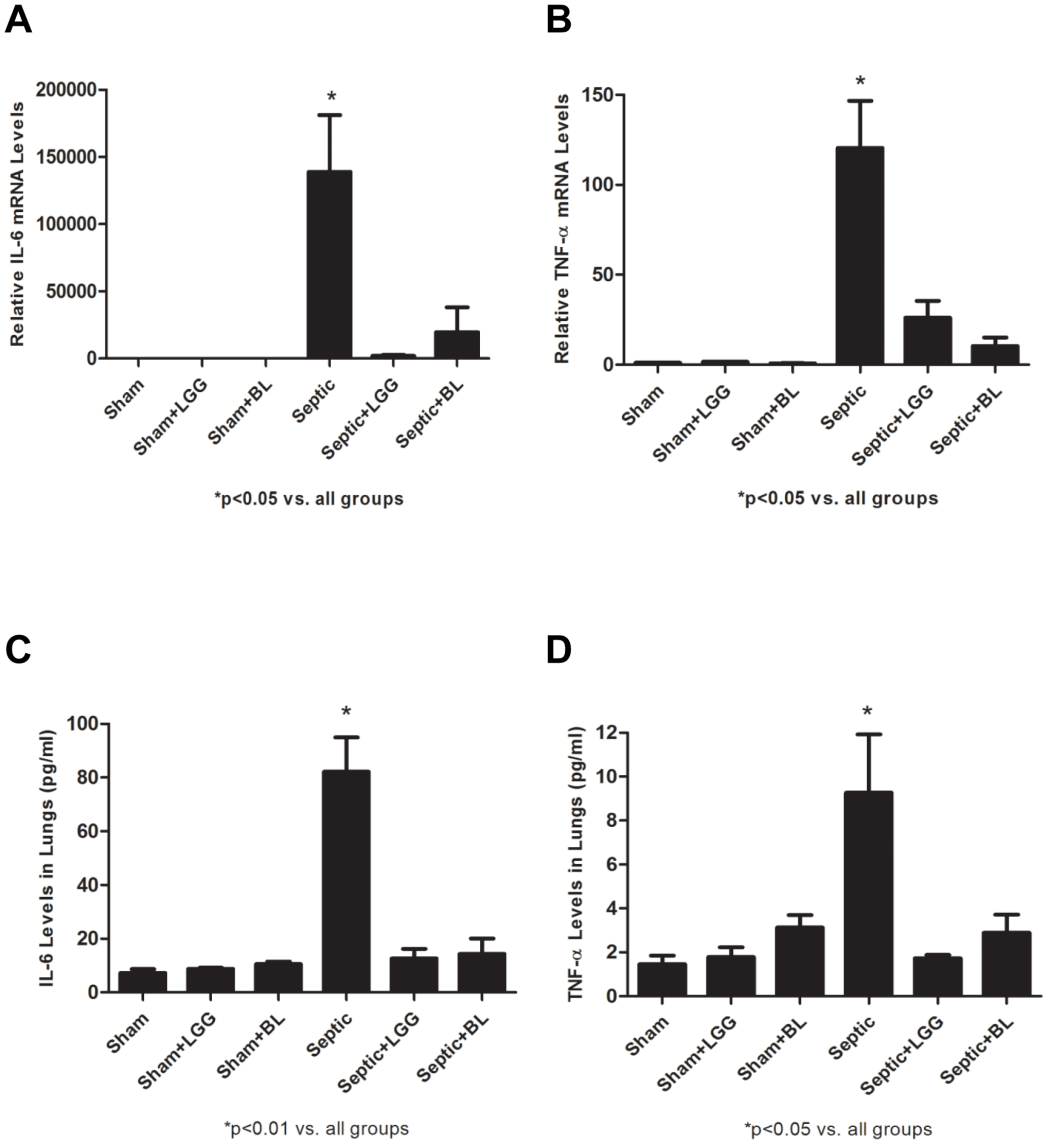
Probiotics attenuate proinflammatory cytokine release in lung 24 hours post CLP. Reverse transcription and real-time PCR assays were performed to quantify steady-state mRNA levels of pro-inflammatory cytokines. (A) IL-6 and (B) TNF-α were significantly increased in the lung of septic animals compare to shams (P<0.05). LGG or BL treatment normalized these levels to shams (P<0.05). Shams n = 4 per group; Septic, Septic+LGG, Septic+BL n = 5 per group. Data are expressed as the mean ± SE. Enzyme-linked immunosorbent assay (ELISA) was used to determine the protein concentrations of IL-6 and TNF-α in the lung. (C) IL-6 and (D) TNF-α were significantly elevated in the lung of septic mice compared to shams (P<0.05). Treatment with LGG or BL prior to CLP led to significantly reduced (P<0.05) levels of both cytokines compared to untreated septic mice. Shams n = 3 per group; Septic, Septic+LGG, Septic+BL n = 4 per group. Data are expressed as the mean ± SE.

To determine the effect of LGG and BL treatment on pro-inflammatory cytokine release in lungs after CLP-induced polymicrobial sepsis, mRNA levels of IL-6 and TNF-α were analyzed by Real-Time PCR and protein levels measured by ELISA. Gene expressions of IL-6 and TNF-α (Figure 3A, B) were significantly increased in lungs of septic animals (P<0.05) and normalized to sham levels in LGG or BL treated mice. Protein levels of IL-6 (P<0.01) (Figure 3C) and TNF-α (P<0.05) (Figure 3D) were also markedly elevated in septic mice and attenuated to sham levels in mice treated with either probiotic strain.

### Probiotics decrease Cox-2 expression and regulate toll-like receptor (TLR) pathway in the lung during sepsis

**Figure 4:**
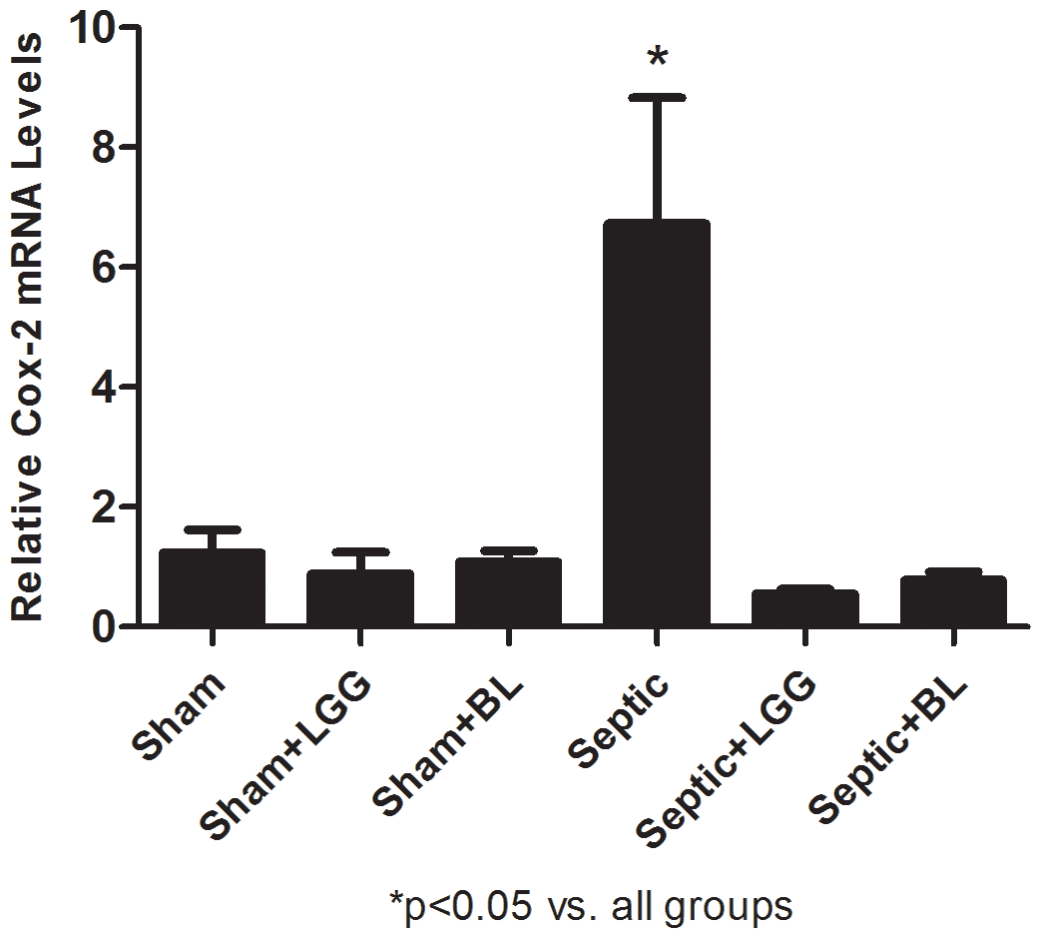
Probiotics downregulate Cox-2 expression in the lung 24 hours post CLP. Reverse transcription and real-time PCR assays were performed to quantify steady-state mRNA levels of Cox-2. Cox-2 was significantly elevated in the septic group compared to sham groups (P<0.05). Treatment with LGG or BL significantly reduced mRNA levels of Cox-2 compared to untreated septic mice (P<0.05). Shams n = 4 per group; Septic, Septic+LGG, Septic+BL n = 4–5 per group. Data are expressed as the mean ± SE.

Cox-2 is rapidly induced in response to cytokines and is elevated at sites of inflammation. Gene expression of Cox-2 was significantly increased in lungs of septic mice (P<0.05) and treatment with LGG or BL significantly decreased (P<0.05) Cox-2 levels to those observed in the lung of sham animals (Figure 4).

**Figure 5:**
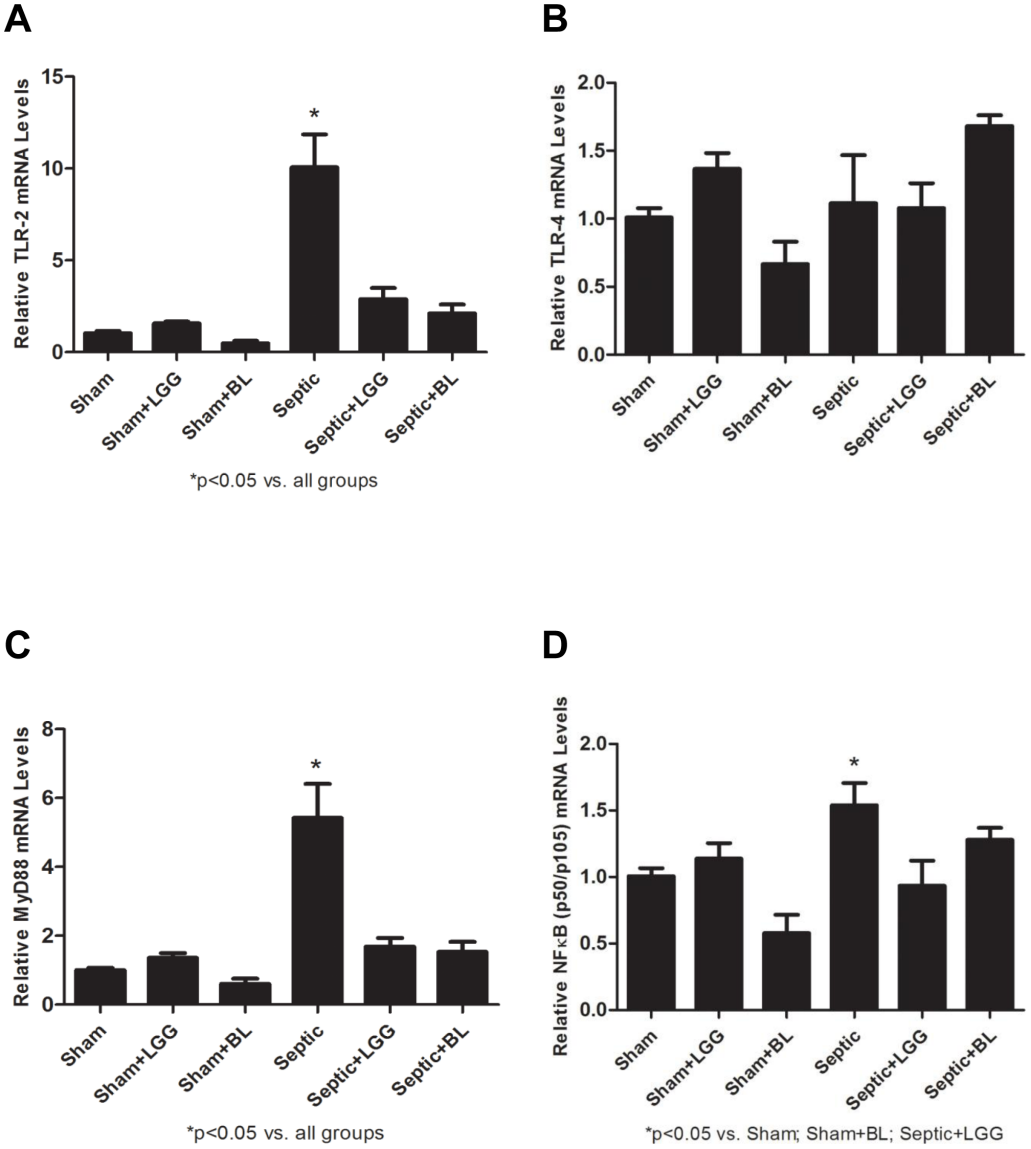
Probiotics regulate Toll-like receptor (TLR) pathway in the lung 24 hours post CLP. Reverse transcription and real-time PCR assays were performed to quantify steady-state mRNA levels of TLR-2, TLR-4, MyD88 and NFΚB (p50/p105). (A) TLR-2 and (C) MyD88 were significantly upregulated in the septic group compared to shams (P<0.05) and significantly downregulated in the lungs of LGG and BL treated septic mice compared to untreated septic mice. (B) mRNA levels of TLR-4 remained unchanged in all groups. (D) NFΚB (p50/p105) was significantly upregulated in the septic group compared to shams (P<0.05) and significantly downregulated in the lungs of LGG treated septic mice compared to untreated septic mice. The levels in BL treated mice were decreased but did not reach statistical significance. Shams n = 5 per group; Septic, Septic+LGG, Septic+BL n = 5–7 per group. Data are expressed as the mean ± SE.

TLRs signal via the MyD88 pathway that includes the NFΚB transcriptional factor, which is a key activator of the cytokines involved in the innate immunity response. MyD88 has an important role in early recruitment of inflammatory cells and in the control of bacterial infection [44]. Gene expression of TLR-2, TLR-4, MyD88 and NFΚB (p50/p105) was analyzed by Real-Time PCR. There was significant increase of TLR-2 MyD88 and NFΚB (p50/p105) in the lungs of septic mice (P<0.05) (Figure 5A, C, D). LGG or BL treatment normalized the levels of TLR-2 and MyD88 to those in shams (Figure 5A, C). NFΚB (p50/p105) was significantly decreased in the lung of LGG treated mice (P<0.05). The levels in BL treated mice were also decreased but did not reach statistical significance (Figure 5D). TLR-4 remained unchanged regardless of treatment (Figure 5B).

## Discussion

This work demonstrates two probiotic strains, *Lactobacillus rhamnosus* GG and *Bifidobacterium longum*, can reduce lung injury and attenuate the inflammatory response in the lungs of weanling mice subjected to CLP.

Not surprisingly, the gut has been identified as an origin and promoter of nosocomial sepsis and multiorgan failure in the critically ill, the major determinant of ICU outcome [45].Critical illness and ICU-based therapies, such as vasopressors and broad spectrum antibiotics, create a hostile environment in the gut and alter the microflora favoring the growth of pathogens. This is in part due to the loss of key beneficial lactic acid bacteria [46] otherwise called probiotics that can inhibit the overgrowth of pathogens by production of bacteriocins, hydrogen peroxide, organic acids, ammonia and by increased competition for adhesion sites on intestinal epithelia [47], [48]. Also, a number of bioactive factors secreted by probiotics, mainly by LGG, have been identified and their effects studied in intestinal injury as well as airway inflammation models [49], [50]. Soluble protein p40 derived from LGG as published by Polk et al preserves barrier function and reduces apoptosis in the colon epithelium in an EGF receptor-dependent manner [51]. A study performed in healthy adults suggests how three different lactobacilli induce differential gene-regulatory networks and pathways in the human mucosa, showing that mucosal responses to LGG involve would healing, IFN response and ion homeostasis [52]. A recent review article provides detailed information on several probiotic strains and their ability to stimulate the immune system including activation of macrophages, natural killer cells, T-lymphocytes and release of cytokines in strain specific, dose dependent manner [53]. Several randomized controlled trials within adult and pediatric populations suggest the use of probiotics as a promising therapy for nosocomial gastrointestinal and respiratory tract infections [10], [11], [12], [13] but there are still many questions to be answered about their mechanisms of action.

From current clinical studies of probiotic therapy, it appears that timing of probiotic administration may be important in their effectiveness with administration early in critical illness potentially being important. [27], [54], [55], [56]. In our mouse model of sepsis, the animals were given LGG or BL immediately before the surgery to better reflect the common clinical setting where a patient presenting with peritonitis could be treated at the time of surgery to attempt to prevent future hospital acquired infections and acute lung injury. Our recently published data describe significant improvement of several outcomes including survival, bacteremia, systemic inflammatory response and intestinal homeostasis with administration of these probiotic strains in this immediate “surgical” timeframe. [36].

The pathophysiology of septic shock syndrome is characterized by hyperactive and dysregulated endogenous inflammatory mediators including cytokines such as IL-6, TNF-α, IL-1β, IL-12 and interferon γ [57], [58]. It has been shown that early attenuation of transcription factor NFΚB activation and cytokine message expression correlates with improved outcome in polymicrobial sepsis [59]. Controlling inflammatory mediated injury to distant organs is a key goal in sepsis to prevent the multiple organ dysfunction syndrome (MODS) which carries quite a high mortality. This is often observed in generalized peritonitis (as studied in our model), which accompanies surgical conditions such as gastrointestinal perforation [58]. Clinical and experimental data support an important role of the lung during the initial stages of the multiple organ dysfunction syndrome (MODS) [60]. The release of pro-inflammatory mediators can cause acute lung injury [61] and it has been reported that levels of pro-inflammatory cytokines such as IL-6 and TNF-α are significantly elevated in the lungs after CLP-induced peritonitis [59], [62]. There are several publications reporting the protective effect of different probiotic strains against bacterial infection. A study done in the rat CLP peritonitis model demostrated a decrease of TNF-α and IL-1β in lungs of animals receiving a prolonged three week pre-treatment with probiotics and overall reduction of acute lung injury was also observed [35]. Racedo at al. used a mouse model of *Streptococcus pneumoniae* infection to evaluate the effect of *L. casei* and found that two days of pre-treatment could beneficially regulate the TNF-α and IL-10 balance, allowing a more effective immune response against infection and modulation the inflammatory response. This was associated with less damage to the lung in this model [63]. In our unique immediate pre-treatment model, we found significantly increased mRNA and protein levels of pro-inflammatory cytokines TNF-α, IL-6 in the lungs of septic mice. Treatment at the time of onset of peritonitis (rather than a prolonged pre-treatment period) with either *Lactobacillus rhamnosus GG* or *Bifidobacterium longum* normalized these cytokine levels to those seen in shams indicating the anti-inflammatory effect of both probiotic strains possibly contributing to better overall outcome.

In general, Cox-2 is not expressed in healthy tissues but is rapidly induced in response to cytokines and is elevated at sites of inflammation and injury [64] and is involved in pathogenesis of sepsis [65]. In mouse CLP model, Cox-2 expression was previously shown to increase in the lungs of septic mice [66], [67], [68], in addition dual inhibition of Cox-2 and 5-LOX successfully attenuated lung injury, reduced MPO activity and improved survival of these mice [69]. As shown in several *in vitro* and *in vivo* models, pathogens induce Cox-2 expression via activated Toll like receptors (TLRs) [39], [40]. TLRs play a central role in the initiation of innate immune responses and in the development of a subsequent pro-inflammatory response, which can lead to inflammation induced organ injury. TLRs are activated by specific microbial ligands leading to an association with TIR domain containing MyD88 factor which mediates a signaling cascade that activates NFΚB factor and results in upregulation of pro-inflammatory cytokines [70]. Markedly increased expression of TLR-2 and TLR-4 in monocytes [71], [72] and leukocytes [73] has been reported in septic patients. In mouse CLP peritonitis models, TLR-2 and TLR-4 expressions were significantly upregulated in hepatic and splenic macrophages [74], in the lungs and liver [75], [76] as well as in the intestine [77] when compared to sham mice. Here we demonstrate that Cox-2, TLR-2, MyD88 and NFΚB (p50/p105) were significantly higher in the lungs of septic mice compared to healthy shams and lower in the lungs of LGG and BL mice. NFΚB (p50/p105) in the lungs of BL treated septic mice showed only a decreasing trend. The expression of TLR-4 in the lungs remained unchanged among all experimental groups, similar to the observations of Williams et al. [75] in a CLP peritonitis model where TLR-4 expression increased at earlier time points but not at 24 hours. We speculate that downregulation of Cox-2 through TLR-2/TLR-4 (via MyD88) in the lungs of *Lactobacillus rhamnosus GG* or *Bifidobacterium longum* treated mice may play a protective role in attenuating inflammation induced lung injury following systemic sepsis and peritonitis.

In conclusion, probiotic therapy with LGG and BL can reduce lung injury following experimental peritonitis and sepsis and is associated with reduced lung inflammatory cell infiltrate and decreased markers of lung inflammatory response activation. Probiotic therapy may be a promising intervention to improve clinical lung injury following systemic infection and sepsis.
